# AGE-RELATED ATTRIBUTIONS OF CHANGE: DETERMINANTS AND CONSEQUENCES

**DOI:** 10.1093/geroni/igz038.1415

**Published:** 2019-11-08

**Authors:** Klaus Rothermund, Clara de Paula Couto

**Affiliations:** 1 FSU Jena, Jena, Thuringen, Germany; 2 FSU Jena, Dept. of Psychology, Jena, Thuringen, Germany

## Abstract

Diehl and colleagues recently introduced the concept of “Awareness of Age-related Change” (AARC; Diehl & Wahl, 2010), emphasizing the need to investigate the subjective experience of one’s own aging. In the Aging-as-Future project (n=1,300 participants, age range 35-85), we separately assessed the experience of changes and attributions of these changes to age. Attributions of changes were driven by a correspondence between the direction of change (gain vs. loss) and negative vs. positive age stereotypes. Importantly, our data also support the assumption that changes interact with age-related attributions in predicting life satisfaction. Specifically, age-related attributions were shown to exacerbate negative effects of losses on well-being.

